# No evidence that religious celibacy confers inclusive fitness benefits: a comment on: ‘Religious celibacy brings inclusive fitness benefits’ Micheletti *et al.* (2022)

**DOI:** 10.1098/rspb.2023.0176

**Published:** 2023-06-28

**Authors:** Lachlan I. von Pein, Kaitlyn T. Harper, Brendan P. Zietsch

**Affiliations:** Centre for Psychology and Evolution, School of Psychology, University of Queensland, Brisbane, Australia

Micheletti *et al.* [1] argue that the seemingly maladaptive cultural practice of religious celibacy may be advantageous through inclusive fitness. They support this claim with three findings from an agropastoralist Buddhist population in western China: (i) men with a monk brother have more children than men whose brothers are not monks (brother analysis); (ii) women with a monk brother-in-law have their first child earlier than women whose brothers-in-law are all not monks (sister analysis); and (iii) men with a monk son have more grandchildren than men with only non-monk sons (father analysis). We believe that these analyses are redundant tests of the same hypothesis and should not be regarded as independent lines of evidence,^1^ although they do require separate rebuttals because of idiosyncratic analytic approaches in each case. Micheletti *et al.* also include an inclusive fitness model outlining the theoretical conditions under which religious celibacy can be adaptive and claim that these conditions are met by their studied population.

In this commentary, we outline inadequacies in the target article's analyses and interpretations that we believe undermine its conclusions. Specifically, we argue that: (i) the significant results of the father analysis are not driven by religious celibacy providing inclusive fitness benefits but rather are due to inappropriate inclusion of participants in the analysis, combined with the wider sample's peculiar demographics; (ii) the brother analysis fails to assess the crucial criterion for selection via inclusive fitness and the studied population is unlikely to satisfy this criterion; (iii) the sister analysis uses a weak, unusual proxy for reproductive success despite a strong, conventional measure being available for a clearer test of the hypothesis; (iv) Micheletti *et al.* have misinterpreted the inclusive fitness model of celibacy with respect to their hypothesis; and (v) mathematical impossibilities reported in the target article's electronic supplementary material raise concerns regarding the general accuracy of Micheletti *et al.*’s analyses.

To avoid confusion, we wish to make clear the peculiar circumstances surrounding access to the target article's full dataset. The publicly available dataset published with the paper excludes numerous variables used throughout the reported analyses. Our request for the full dataset received no response from the authors and was eventually denied via the journal, with ethical concerns regarding participant anonymity cited as the reason. Data omitted include control variables (preventing us from replicating any of Micheletti *et al.*'s findings precisely) as well as several predictor and outcome variables (preventing us from replicating three of Micheletti *et al.*'s seven findings in any capacity; see table 1 for details). Our inability to replicate many of Micheletti *et al.*'s findings, let alone conduct the additional analyses we deem appropriate, presents a dilemma between fulfilling the ethical requirements of Micheletti *et al.*'s study and maintaining the integrity of the scientific process through transparency of analysis and potential for independent critique. To resolve this conflict, we have been invited by *Proceedings of the Royal Society B* to ‘write a Comment explaining [our] interest in the paper and the analysis [we] wish to conduct’ to which ‘the authors have agreed to respond with a Reply paper, where they will run the requested analyses and provide the data’.

**Table 1.** Variable availability in public Micheletti *et al.* datasets.

	brother analysis		sister analysis		father analysis	
	variable	publicly available	variable	publicly available	variable	publicly available
main variables	number of children	yes	number of children	yes	number of grandchildren	yes
	whether each individual has a brother but no monk brothers	yes	whether each individual has a monk brother	yes	whether each individual has a monk son	yes
	whether each individual has a monk brother	yes	age at first birth	no	number of sons	no
	whether each individual is a first born son	no	whether each individual has a monk brother-in-law	no		
control variables	birth cohort	yes	birth cohort	yes	birth cohort	yes
	village	yes	village	yes	village	yes
	number of sisters	no	number of brothers	no	number of offspring	no
	household wealth	no	number of sisters	no	household wealth	no
	distance to county capital	no	household wealth	no	distance to county capital	no
			distance to county capital	no		

Before addressing our key criticism of the father analysis, we highlight its ambiguous description. Micheletti *et al.* initially state that ‘men with at least one child who have a monk son have 1.15 times more grandchildren than men with only lay sons'. They then state that ‘the effect remains significant when restricting the analyses to men with at least one son’. The first statement suggests the first comparison only considers men with at least one son, but the additional restriction described in the second comparison implies this is not the case. Our assumption—which we ask Micheletti *et al.* to verify—is that the first comparison actually includes all men with at least one child, whereas the second comparison restricts this sample to only include men with at least one son (i.e. removes all men with only female children).

Under this assumption, we argue that the father analysis's findings are not driven by inclusive fitness benefits of religious celibacy; rather, they are due to inappropriate inclusion of a subset of men who cannot have a monk son and who, incidentally, also have substantially diminished fertility. Our explanation of this confound is as follows. Regarding male children, Micheletti *et al.* state that ‘first borns only rarely become monks’, which implies that, for the most part, monks are only produced by families with at least two sons. Applying this inference to the father analysis's second comparison (whose sample contains men with at least one son), it appears that this analysis includes numerous men with only one son, who are, therefore, very unlikely to father a monk. The inclusion of these superfluous men introduces a significant confound for two reasons: (i) the three-child limit imposed on the studied population implies that these superfluous men, who have only one son, presumably have more daughters on average than the rest of the men considered in this second comparison of the father analysis (who all have at least two sons and are, therefore, more likely to have a monk son)^2^ and (ii) the average fertility for women in this sample is around half that of men,^3^ implying a strong correlation between having daughters and having fewer grandchildren.^4^ That is, by inappropriately including men with one son in their father analysis, we believe Micheletti *et al.* have unknowingly increased the average number of female children for the group of men without monk sons and, in doing so, have artificially diminished this group's average number of grandchildren (controlling for number of children).

Crucially, the father analysis results suggest that this confound is driving this section's significant findings. While the *p*-value of the father analysis's first comparison is highly significant at 0.005, we note that the second comparison's *p*-value is much less significant at 0.037. If these comparisons differ only in that the second comparison restricts the sample of the first by excluding men with only female children, this difference in *p*-value would reflect the confound outlined above. Furthermore, the marginal *p*-value of the second comparison suggests that, had men with only one son been appropriately excluded from this analysis (thereby increasing the average number of grandchildren for the group of men without a monk son), this *p*-value could exceed the conventional 0.05 significance threshold, such that there would be no evidence for the hypothesized effect. We ask that Micheletti *et al.* evaluate the validity of this argument by performing the father analysis on men with at least two sons. We are unable to perform this analysis ourselves due to the provided dataset missing the following variables: number of offspring, number of sons, and relevant control variables.

We now turn to the brother analysis, where Micheletti *et al.* report that ‘men with a monk brother have 1.75 times more children than men whose brothers are not monks'. We argue that this result alone does not support Micheletti *et al.*'s claim that religious celibacy provides inclusive fitness benefits. Supporting this claim would require evidence that celibacy offers a reproductive benefit to one's close kin *and* that this benefit is sufficiently great to outweigh the inclusive fitness cost incurred by the celibate forfeiting all reproductive potential. It is entirely possible—and, as we argue below, very probable—that having a monk brother is associated with men having 1.75 times more offspring, as reported by the brother analysis, but for this behaviour to still be maladaptive. Micheletti *et al.* fail to address this possibility, so the brother analysis does not provide evidence that religious celibacy confers inclusive fitness benefits.

The unavailability of Micheletti *et al.*'s data prevents us from conducting our own accurate assessment of the brother analysis. However, the limited information available indicates that the reported reproductive advantage is insufficient to allow selection of religious celibacy. If men with a monk brother have 1.75 times more children, then for families with only two sons, those with one celibate and one non-celibate son have fewer children on average (1.75 times the average male fertility) than those with two non-celibate sons (whose two sons combined have 2 times the average male fertility). While this figure of 1.75 times more children also implies that having a monk son is beneficial for families with three or more sons, the three-child limit imposed on the studied population means that this advantage applies only to families with three sons and no daughters. Within three-child families, families with two sons and one daughter should outnumber families with three sons approximately 3 : 1,^5^ and so families for whom religious celibacy is advantageous will be in a pronounced minority. In short, even if we make the implausible assumption that all families have three children, the results of the brother analysis suggest that committing a son to celibacy would be disadvantageous to most families. While we acknowledge this argument relies on a number of simplifying assumptions (a limitation imposed on us due to lack of data access), it does demonstrate that the brother analysis as presented provides no evidence that religious celibacy is adaptive. Should Micheletti *et al.* maintain that the brother analysis does support their hypothesis, they would need to demonstrate why—for example, by explaining how peculiarities of their sample overturn the apparent insufficiency of the reproductive advantage as outlined above.

We now turn to the sister analysis which reports that, of women with at least one brother, women with and without a monk brother have similar numbers of children and, therefore, having a monk brother is not reproductively advantageous to women. This result is contrasted against the finding that women do receive a ‘reproductive advantage’ by having a monk brother-in-law, in the form of a slightly earlier age at first birth. Our issue with this section of the target article is twofold. First, it is unclear to us how these results support the conclusion Michelleti *et al.* draw from them: that ‘the benefits obtained by men from their monk brothers is not accrued through polygamy, but is achieved through earlier reproduction of their wives'. Second, we do not understand why Micheletti *et al.* use a direct, conventional proxy of reproductive success (number of children) in the first sister comparison (and all other analyses) but then switch to a weak, unusual one (age at first birth) for the subsequent sister-in-law comparison. If the second comparison had assessed number of offspring and found an advantage for women with a monk brother-in-law, and that this benefit outweighed the associated costs, it would support Micheletti *et al.*'s claims and the age of first birth of these women would be irrelevant. Similarly, if it were found that women with and without a monk brother-in-law have similar numbers of children, it would indicate that having a monk brother-in-law is unrelated to a woman's fitness, regardless of age of first birth. We are unable to perform our recommended analysis because there was no provided dataset of women with and without a monk brother-in-law. To address these considerations, Micheletti *et al.* could explain: (i) why monk sisters not receiving a reproductive benefit and/or monk sisters-in-law having children slightly earlier implies ‘the benefits obtained by men from their monk brothers is not accrued through polygamy’ and (ii) why they switched proxy measures for reproductive success throughout the sister analysis and to provide results of the sister (brother-in-law) analysis using number of offspring as the outcome variable.

Regarding the inclusive fitness model of celibacy, we argue that Micheletti *et al.* have misinterpreted the conditions under which the model allows for the selection of celibacy. The target article's model dictates that it is advantageous for ‘parents to commit a son to lifelong celibacy whenever the benefit to the lay son outweighs the cost to the monk’: a condition formally expressed by the inequality

$$c(\bar{x})<b(\bar{x}).$$

(Equation (3.4) in the target article; $c(\bar{x})$ denotes the reproductive cost incurred by the celibate; $b(\bar{x})$ denotes the reproductive benefit provided to the celibate's brothers.) We believe this claim is valid and mirrors our previously outlined requirements to establish that religious celibacy offers inclusive fitness benefits. However, Micheletti *et al.* then claim that with their inclusive fitness model, they have shown that ‘men can be favoured by selection to be celibate … when having a monk brother makes men more competitive, leading to higher reproductive success'. This statement (that having a monk brother must make men more competitive, i.e. $b(\bar{x})>0$) is *not* the criterion outlined by the model (that the benefit to the lay son must outweigh the cost to the monk, i.e. $c(\bar{x})<b(\bar{x})$). Contrary to Micheletti *et al.*'s interpretation, we argue the model's conditions are probably not met by Micheletti *et al.*'s studied population, given our above argument as to why the brother analysis's reported reproductive benefit for monk brothers does not appear to outweigh the cost to the monk.

A further point regarding Micheletti *et al.*'s model is that it assumes that variation among individuals' propensity to send a son to the monastery is ‘inherited as if genetically controlled’. However, the authors also say they ‘are not claiming that this trait is controlled by a single gene or even that it is influenced by genetics at all. The trait could be—and most likely is—culturally transmitted. The aim of the model is to better understand to what extent it might have been shaped by inclusive fitness interests.' To be clear, genetic and cultural transmission cannot be assumed to be the same or even similar; therefore, without careful, rigorous justification, a model assuming the former cannot be used to draw conclusions about how the latter has shaped a trait. The authors provide no such justification, yet conclude, for example, that their model suggests ‘this cultural practice has been shaped heavily by the inclusive fitness interests of the monks' parents’.

As a final note, we also wish to point out mathematical impossibilities in Micheletti *et al.*'s electronic supplementary material tables. Table S11 (electronic supplementary material) states that of the 929 women considered in the relevant analysis, the average age at first birth is 20.87. After partitioning this sample into two groups of 91 and 838 women, it is reported that the average age at first birth for these groups is 20.9 and 21.4, respectively. The total mean cannot be less than the means of both the constituent subgroups. Furthermore, table S1 (electronic supplementary material) reports that of the 934 men considered in the brother analysis, 646 men do not have any brothers. However, table S5 (electronic supplementary material) reports that once men with older brothers are excluded from this sample, reducing it from 934 to 780, 615 men do not have any brothers. Given that all 646 men in the original sample of 934 do not have any brothers and, therefore, do not have any older brothers, these men should also be included in the sample of 780. That is, there should be exactly 646 men in the sample of 780 who do not have any brothers. This discrepancy implies that 31 men without brothers have been excluded from the analyses presented in electronic supplementary material, S5–S8. There are other sample size inconsistencies in tables S1 and S5 (electronic supplementary material), but we have omitted these explanations for brevity. Due to lack of data access, we cannot determine the impact, if any, of these errors on the target article's findings.

In our view, the criticisms presented here invalidate Micheletti *et al.*'s argument that religious celibacy brings inclusive fitness benefits. The significant finding of the father analysis appears to be confounded due to inappropriate choice of sample, the results of the brother analysis suggest, if anything, that religious celibacy is indeed maladaptive, and the sister analysis uses a dubious measure of fitness. Furthermore, if interpreted correctly, their inclusive fitness model of religious celibacy suggests that the studied population does not meet the conditions under which celibacy can be adaptive. The burden of establishing their claim rests with the authors. Our suggested analyses, which we cannot conduct without data access, would clarify these issues; we believe they would underscore the need to revise the claim that religious celibacy brings inclusive fitness benefits.

## Data Availability

This article has no additional data.

## Footnotes

1 For example if men with a monk son had more grandchildren than men with only non-monk sons (father analysis), then it would necessarily be true that men (and/or women) with a monk brother have more children than men (and/or women) whose bothers are not monks (brother/sister analysis).

2 To clarify, the three-child limit means that men with at least two sons can have either 0 or 1 daughters, whereas men with only one son can have either 0, 1 or 2 daughters. We, therefore, expect that the latter group has a greater female offspring rate than the former, but limited data access prevents us from verifying this point.

3 Electronic supplementary material, table S1, reports that the 934 men considered in the brother analysis (i.e. all living, non-monk men born from 1961 to 2000) have an average fertility of 1.53, implying these 934 men have a combined 1429 children. Electronic supplementary material, table S1, also reports that men in this sample have on average 0.14 monk brothers, implying these 934 men combined have 131 monk brothers. From this, we obtain that Micheletti *et al.*’s sample contains 1065 men born from 1961 to 2000, and these men have a combined 1429 children, implying an average fertility of 1.34. Electronic supplementary material, table S9, reports that women considered in the sister analysis (i.e. all living women with at least one brother born from 1961 to 2000) have an average fertility of 0.70. Note that since the second comparison of the father analysis only considers men with at least one son, the fertility rate of women in electronic supplementary material, table S9, i.e. women with at least one brother, is precisely the female fertility rate relevant to the confound we present.

4 This correlation is noteworthy in and of itself. Given that each child has exactly one mother and one father, male and female fertilities across a population must be equal. The fact that Micheletti *et al.*’s sample violates this requirement, in our view, indicates that something very unusual has occurred in their sampling method.

5 For a three-child family, there is exactly one way to achieve three sons (M, M, M) and three ways to achieve two sons and one daughter (M, M, F; M, F, M; F, M, M).
